# Origin of core-to-core x-ray emission spectroscopy sensitivity to structural dynamics

**DOI:** 10.1063/4.0000022

**Published:** 2020-07-06

**Authors:** Morgane Vacher, Kristjan Kunnus, Mickaël G. Delcey, Kelly J. Gaffney, Marcus Lundberg

**Affiliations:** 1 Department of Chemistry-Ångström Laboratory, Uppsala University, SE-751 20 Uppsala, Sweden; 2 Laboratoire CEISAM–UMR CNRS 6230, Université de Nantes, 44300 Nantes, France; 3 PULSE Institute, SLAC National Accelerator Laboratory, Stanford University, Menlo Park, California 94025, USA; 4 Institute of Physics, University of Tartu, W. Ostwaldi 1, EE-50411 Tartu, Estonia

## Abstract

Recently, coherent structural dynamics in the excited state of an iron photosensitizer was observed through oscillations in the intensity of K*α* x-ray emission spectroscopy (XES). Understanding the origin of the unexpected sensitivity of core-to-core transitions to structural dynamics is important for further development of femtosecond time-resolved XES methods and, we believe, generally necessary for interpretation of XES signals from highly non-equilibrium structures that are ubiquitous in photophysics and photochemistry. Here, we use multiconfigurational wavefunction calculations combined with atomic theory to analyze the emission process in detail. The sensitivity of core-to-core transitions to structural dynamics is due to a shift of the minimum energy metal–ligand bond distance between 1*s* and 2*p* core-hole states. A key effect is the additional contraction of the non-bonding 3*s* and 3*p* orbitals in 1*s* core-hole states, which decreases electron–electron repulsion and increases overlap in the metal–ligand bonds. The effect is believed to be general and especially pronounced for systems with strong bonds. The important role of 3*s* and 3*p* orbitals is consistent with the analysis of radial charge and spin densities and can be connected to the negative chemical shift observed for many transition metal complexes. The XES sensitivity to structural dynamics can be optimized by tuning the emission energy spectrometer, with oscillations up to ±4% of the maximum intensity for the current system. The theoretical predictions can be used to design experiments that separate electronic and nuclear degrees of freedom in ultrafast excited state dynamics.

## I. INTRODUCTION

Transition-metal complexes are key components in many photocatalytic processes. An area of intense interest is the conversion of solar to chemical energy stored in fuels.^1,2^ A solar fuel system consists of three major components, a photosensitizer, a catalyst for reductive fuel formation, and a catalyst for the oxidation of an electron donor. The photosensitizer absorbs a photon to form a long-lived valence-excited state, from which an electron can be transferred to the fuel-forming reaction. As many photosensitizers are metal complexes, detailed characterization of charge-transfer excited states of such systems is not only of interest for fundamental research but also directly applicable to light-harvesting applications.^3,4^

To understand the efficiency of the charge transfer process, the electronic and structural dynamics along the excited state relaxation pathway must be probed. X-ray spectroscopy is ideally suited for this task as it is possible to selectively probe a specific site in a complex environment. With the use of laser pumps in combination with ultrafast x-ray probes, it is also possible to study processes on very short time scales.^5^ For ultrafast time-resolved experiments, nonresonant x-ray emission spectroscopy (XES), both K*α* (2*p* → 1*s*) and K*β* (3*p* → 1*s*), has been successfully used to study transitions between electronic states.^6–11^ This relies on the sensitivity of XES spectra to the metal oxidation state and spin multiplicity.^12–14^ Complementary to XES, structural dynamics can be probed by elastic x-ray scattering.^15–22^ XES and scattering can also be detected simultaneously, making it possible to probe the connection between electronic and structural dynamics.^7,10,23^

The most commonly used photosensitizers are ruthenium coordination complexes. However, for large-scale applications, catalysts based on widely abundant non-toxic base metals such as iron are desirable.^24–26^ Iron carbenes have attracted considerable interest due to the possibility to design systems with extended excited state lifetimes and high electron injection efficiencies.^27,28^ This is due to the strong *σ*-donor binding associated with carbene ligands, which leads to a destabilization of the metal-centered (MC) states that are involved in rapid recombination. Instead, this gives long lifetimes for the metal-to-ligand charge-transfer (MLCT) states, from which electrons can be transferred.

**FIG. 1.**
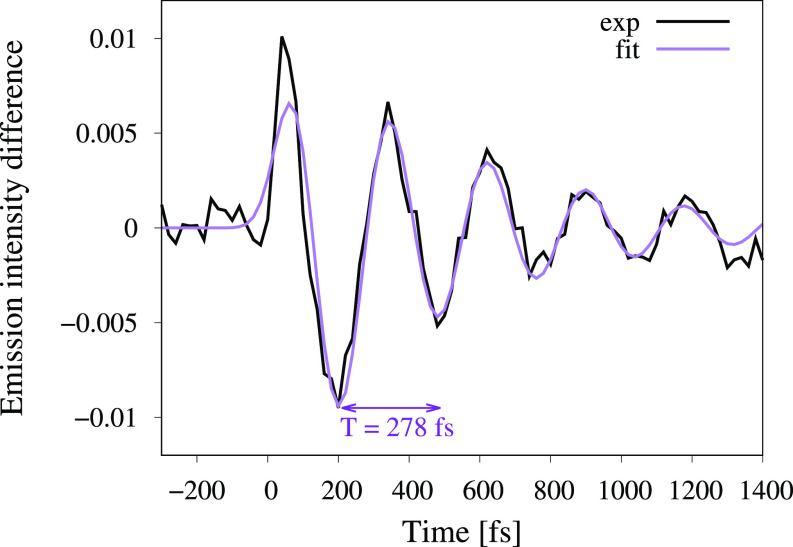
Femtosecond oscillations in the K*α* emission intensity upon photoexcitation of [Fe^*II*^(bmip)_2_]^2+^, from Ref. 23. The emission intensity was measured at a fixed emitted photon energy of 6404.3 eV corresponding to the maximum of the K*α*_1_ XES peak of the electronic ground state (using a spherically bent Ge(440) crystal in Rowland geometry). The purple curve corresponds to the analytical expression I(t)=(p*A)(t) with $A(t)=\text{cos}\;(\frac{2\pi }{T}t)\times \text{exp}\;(-\frac{t}{\tau_{\mathrm{damp}}})$, where the parameters *T* and *τ_damp_* were fitted to the experimental data and *p* is a convolution function taking into account the indirect population of the ^3^MC state (for further details, see the supplementary material in Ref. 23).

A prominent example of an Fe carbene photosensitizer is [Fe^II^(bmip)_2_]^2+^ [bmip = 2,6-bis(3-methyl-imidazole-1-ylidine)-pyridine]. The COOH-functionalized derivate of this complex can inject electrons into a TiO_2_ substrate with very high efficiency.^28^ This complex was recently probed using the combination of femtosecond K*α* and K*β* XES with x-ray solution scattering (XSS).^23^ To be more precise, K*α* XES was measured at a fixed emitted photon energy, corresponding to the maximum of the K*α*_1_ XES peak, while the K*β* signal was integrated over the full spectral range. These measurements show temporal oscillations in the XES and XSS difference signals with the same 278 fs period oscillation (see Fig. 1). By combining the results from these two experiments, the oscillatory signal is assigned to a vibrational wavepacket along a Fe-ligand stretching mode on a triplet metal-centered (^3^MC) excited state surface. This was the first observation of purely structural dynamics with core-to-core x-ray emission.

Understanding the origin of this structural sensitivity is important for further development of femtosecond time-resolved XES methods and, we believe, generally necessary for interpretation of XES signals from highly non-equilibrium structures that are ubiquitous in photophysics and photochemistry. Core-hole spectroscopy can probe metal–ligand interactions due to the strong interactions between core and valence electrons, but core-to-core XES is largely considered an atomic phenomenon because transitions occur between two core orbitals that are not directly involved in metal–ligand binding. The sensitivity of K*α* (and K*β*) XES to nuclear wavepacket dynamics can therefore be considered unexpected and requires a detailed explanation. A better understanding of this phenomenon can also be useful in the interpretation of other experimental data that show time-resolved oscillations. As first shown in Ref. 23, this sensitivity appears to be a result of the relative displacement (vibronic coupling) of the associated core-ionized potential energy surfaces. However, the detailed physical origin of this vibronic coupling and its dependence on the chemical bonding is still not established. In this work, we use the example of [Fe^II^(bmip)_2_]^2+^ to explain the structural sensitivity of the core-to-core x-ray emission spectra and discuss in detail the effect of various metal–ligand electronic interactions.

Electronic and geometric structures are connected to XES spectra through multiconfigurational calculations based on the *ab initio* restricted active space (RAS) approach.^29,30^ This method includes a formally correct coupling of the multiple open shells created during the x-ray process, while at the same time, it directly describes metal–ligand interactions in a molecular orbital picture.^31–33^ We recently introduced efficient algorithms to control the occupation numbers of multiple core orbitals, which makes it possible to simulate core-to-core XES processes.^34,35^ The goal of this contribution is to convert the multiconfigurational results into an intuitive physical explanation for the shift by connecting it to a more straightforward atomic model.^36,37^

## II. COMPUTATIONAL DETAILS

The geometries for the ground state (GS) and ^3^MC energy minima were obtained from density-functional theory (DFT) PBE0/6-311G(d,p) optimizations, including the MeCN solvent through the polarizable continuum model.^38^ Two additional geometries were created on each side of the ^3^MC energy minimum by changing the geometry along the GS—^3^MC distortion coordinate, with Fe-ligand distances changing on average by −0.01 Å for ^3^MC short and by +0.025 Å for ^3^MC long. These two geometries represent the extreme turning points of the average oscillation of the Fe-ligand bond length according to wavepacket quantum dynamics simulations and XSS measurements.^23^ The GS minimum structure belongs to the D_2_ point group, but as the iron is six-coordinated, O_*h*_ point group labels will be used to describe the metal orbitals. The Cartesian coordinates of the four structures are given in the supplementary material.

K*α* emission spectra were calculated using the RAS approach in the OpenMolcas package (version v18.0.o180105–1800).^39^ All calculations use the ANO-RCC basis set with polarized double-zeta contraction (ANO-RCC-VDZP)^40,41^ and the atomic compact Cholesky decomposition.^42^ Orbital optimizations were performed using the state-average RAS self-consistent field (SCF) formalism,^30^ performed separately for each spin multiplicity. The sensitivity of the results with respect to adding dynamic correlation was tested at the GS and ^3^MC minimum geometries through second-order perturbation theory with the RASPT2 method (see Sec. I in the supplementary material for more details).^43^

**FIG. 2.**
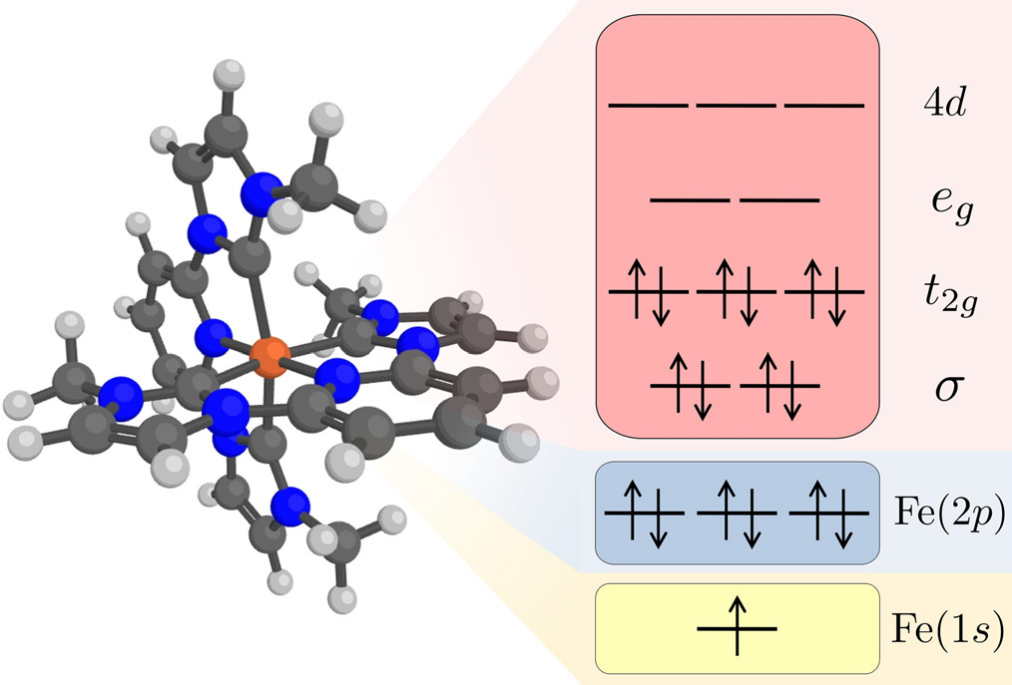
Restricted active space used in the calculations of K*α* x-ray emission. The electron configuration represents the 1*s* core-hole intermediate of the ground state.

**FIG. 3.**
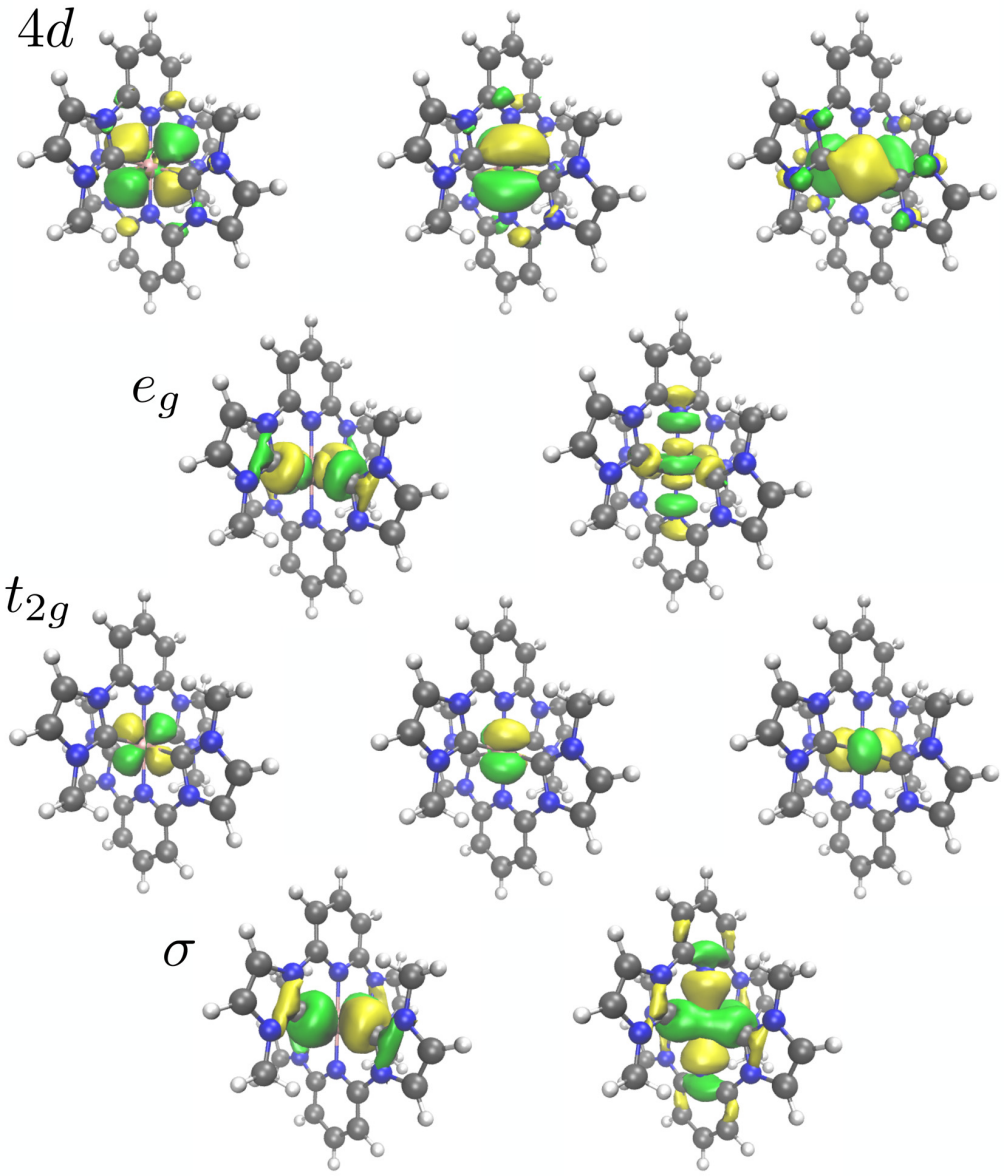
Active orbitals in the ground state placed in the RAS2 space: *σ*, $t_{2g}$, *e_g_*, and 4*d* (the radial nodes of the latter are difficult to see here).

The valence active space consisted of 10 electrons distributed in 10 orbitals, five Fe 3*d* dominated orbitals and five corresponding correlating orbitals (see Fig. 2). These orbitals can be classified in two filled Fe-ligand *σ*-bonding orbitals together with two metal-dominated *e_g_* orbitals forming σ* orbitals with the ligand and three filled metal-dominated non-bonding $t_{2g}$ orbitals together with three empty orbitals that can correlate with the filled $t_{2g}$ orbitals (see Fig. 3). These are metal 4*d* orbitals that describe the double-shell effect.^44^ These orbitals were placed in the RAS2 space, where all possible excitations were allowed.

The Fe 1*s* orbital was placed in the RAS3 space, allowing for a maximum of two electrons, while the three Fe 2*p* orbitals were placed in the RAS1 space, allowing for a maximum of one hole (see Fig. 2). To model the core-ionized states and ensure the hole stayed in the core orbitals instead of higher-lying orbitals, the core hole orbitals were kept frozen in the RASSCF optimizations of the 1*s* and 2*p* core-ionized states. To avoid calculating all lower-lying excited states, we have used a projection operator technique, along the lines of the core-valence separation technique, which sets the configuration interaction (CI) coefficients of all unwanted configurations with doubly occupied core orbitals to zero.^35^

To select relevant spin multiplicities, the spin-selection rules of the electric dipole transitions (ΔS=0) and the selection rules for the spin–orbit operator (ΔS=0,±1) were considered. The valence electronic states were calculated with the singlet and triplet spin multiplicities, while the core-ionized states were calculated with doublet and quartet spin multiplicities. For the ^3^MC states, the 1*s* core hole was averaged over 9 doublet and 6 quartet states. For the 2*p* core-hole states, 50 doublet and 18 quartet states were used. To be able to converge the CI calculations, we have used our recently developed CI algorithm designed for use with a large number of states, which puts a cap on the number of CI vectors and then dynamically allocates more CI vectors to states that have not yet been converged.^35^

Spin–orbit coupled states were obtained using a Douglas–Kroll–Hess Hamiltonian and atomic mean field integrals^45,46^ by the RAS state-interaction (RASSI) approach.^47,48^ In addition to calculate the spin–orbit coupling, the RASSI method is also used to calculate electric dipole oscillator strengths. The photoionization process for the closed-shell ground state has been calculated using the Dyson-orbital formalism, recently implemented for closed-shell species in OpenMolcas.^39^ For triplet initial states, a statistical ratio of 4:2 for quartet and doublet core-excited states has been assumed.

For comparison to experiment, RAS spectra were broadened with a Gaussian of 0.39 eV half-width-at-half-maximum (HWHM) and Lorentzian function of 0.81 eV HWHM.^23,49^ The spectrum for the GS geometry was shifted by 17.35 eV to align with the experimental K*α*_1_ emission maximum, and the intensity was scaled by 1/0.067 so that the GS maximum intensity is 1. The same energy shift and intensity scaling factor were then used for all calculated spectra. The RASSCF wavefunctions were analyzed by examining the orbital composition and by calculating the radial electron density using the Multiwfn program.^50^

Calculations of atomic ions have been performed using the CTM4XAS interface to an atomic multiplet code.^51,52^ This makes it possible to connect the multiconfigurational wavefunction results with this commonly used method to describe x-ray spectra. In this method, energies are obtained from Hartree–Fock calculations, with electron correlation approximated by scaling down the electron–electron repulsion integrals to 80%.

## III. RESULTS AND DISCUSSION

The first part of this section analyzes the simulated K*α* XES spectra in detail, with focus on different contributions from intermediate core-ionized states. The emission from these states is then analyzed in detail, with regard to their sensitivity to the geometric structure. In the next step, spin and charge densities in the important core-excited states are analyzed in order to connect the changes in emission spectra to metal–ligand bonding characteristics and the chemical shift. Finally, the general requirements to observe structural dynamics are outlined and discussed in relation to other types of complexes and emission processes.

### A. X-ray emission spectra

**FIG. 4.**
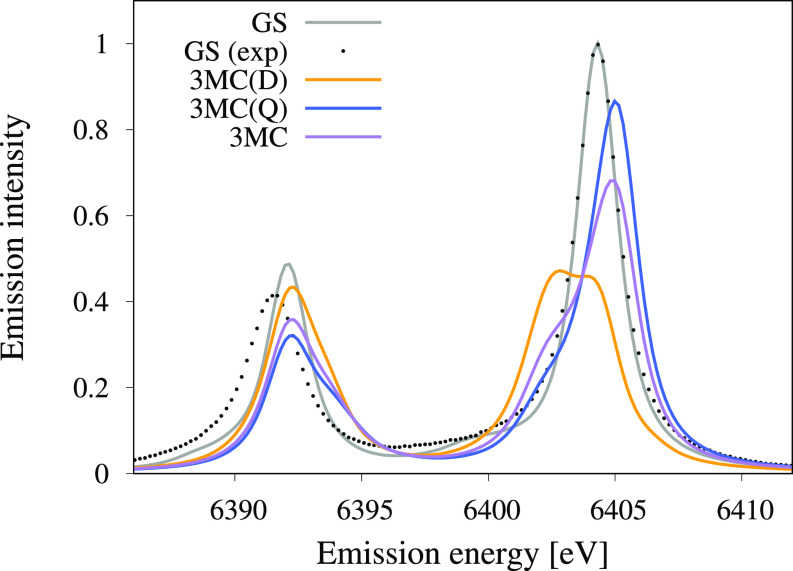
Calculated K*α* emission spectra for the ground state (GS) and for the lowest-energy ^3^MC state via a doublet ^3^MC(D) and a quartet ^3^MC(Q) intermediate state. The ^3^MC spectrum is obtained from a 4:2 ratio of ^3^MC(Q) and ^3^MC(D). All spectra are calculated at the same geometry and the ground state minimum energy structure. The experimental spectrum measured for the GS is indicated with black dots.

Experimentally, the ground state has a K*α*_1_ intensity maximum at 6404.3 eV, with the K*α*_2_ maximum at 12.8 eV lower in energy. From the closed-shell valence configuration of the ground state, 1*s* photoemission only reaches one doublet intermediate state, and no significant shake-up transitions are observed. From this state, the only important transitions are those to states with a 2*p* hole coupled to the closed-shell valence. These final states are split by 0.1 eV due to deviations from the 2*p*-degeneracy in formal O_*h*_ symmetry and then further by the 2*p* spin–orbit coupling, leading to the simulated spectrum in Fig. 4 (gray line). After aligning the energy of the K*α*_1_ peak to experiment, the agreement is very good, as can be expected from such a relatively simple process. The energy of the K*α*_2_ peak is slightly too high, which is likely due to limitations in the description of the strong 2*p* spin–orbit coupling.^53^

The ^3^MC valence excited states reached after photoexcitation have a formal $t_{2g}^{5}e_{g}^{1}$ orbital occupation, which gives rise to six different states (see Fig. S1 in the supplementary material). 1*s* ionization from any of the six ^3^MC states leads to doublet and quartet core-ionized intermediate states. Figure 4 presents here the K*α* emission for the lowest-energy ^3^MC state, via the doublet ^3^MC(D) and quartet ^3^MC(Q) 1*s* core-ionized intermediate states. There are significant differences in the emission spectra of different intermediate states. The ^3^MC(Q) spectrum shows a significant blueshift of the emission peak, while the ^3^MC(D) spectrum shows a significant redshift of the emission peak. As the quartet contribution dominates, the ^3^MC emission spectrum is blue shifted relative to the GS. The ^3^MC contributions are generally broad, especially for the ^3^MC(D) state. All of this leads to a significant drop in intensity at the GS K*α* maximum energy when measuring the ^3^MC emission. To test the robustness of the results with respect to the state character (or the valence electronic configuration), we have also calculated the emission spectra for the two following higher-energy ^3^MC states; the data are gathered in Fig. S2 in the supplementary material. Although there are some differences between the three ^3^MC states, these are much smaller than the differences between the contributions from intermediate states of different spin multiplicities.

**FIG. 5.**
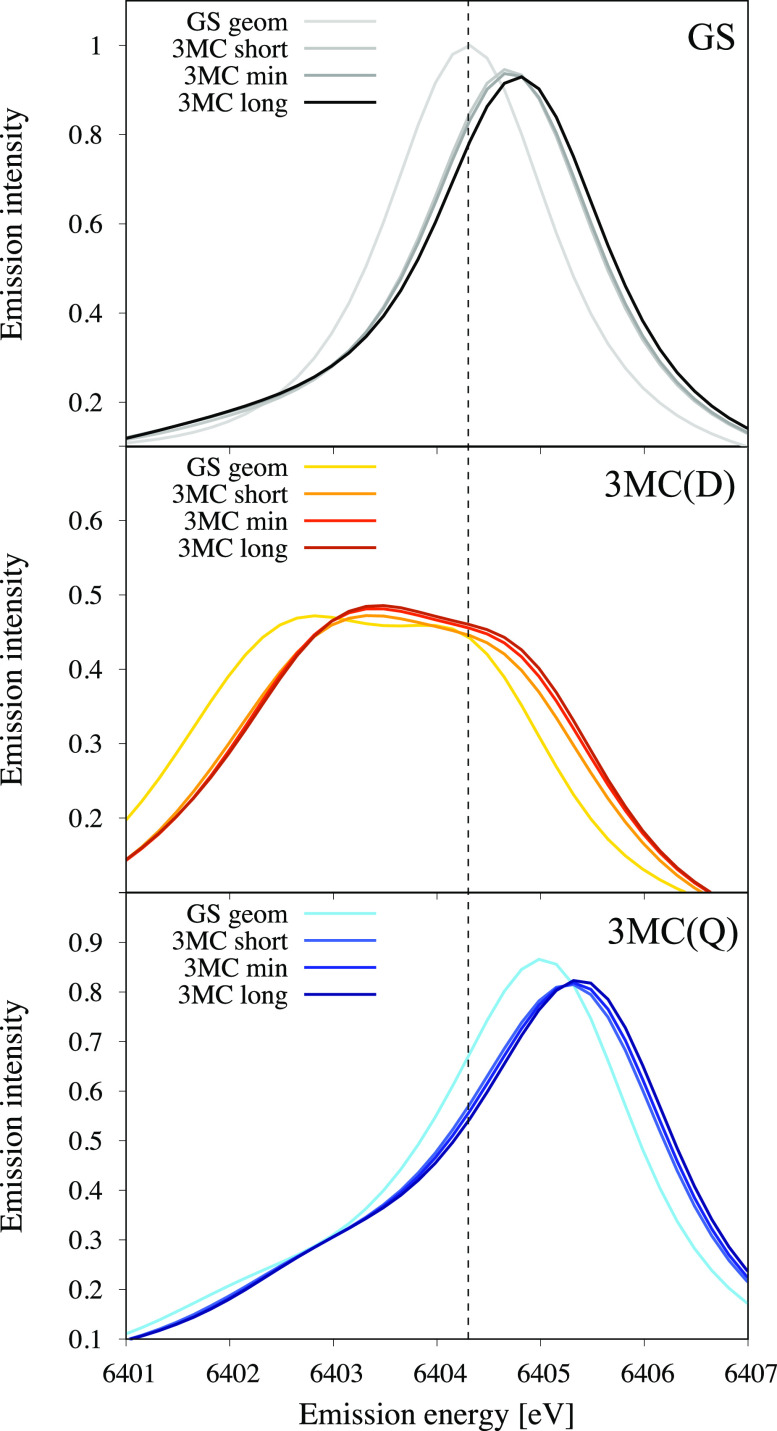
Calculated K*α* emission spectra for the GS (top), ^3^MC(D) (middle), and ^3^MC(Q) (bottom) at different geometries: the GS minimum “GS geom,” the lowest ^3^MC minimum “^3^MC min,” and two other geometries on each side of the ^3^MC energy minimum labeled “^3^MC short” and “^3^MC long.” The vertical dashed line indicates the energy of the GS emission maximum at the GS minimum geometry.

**FIG. 6.**
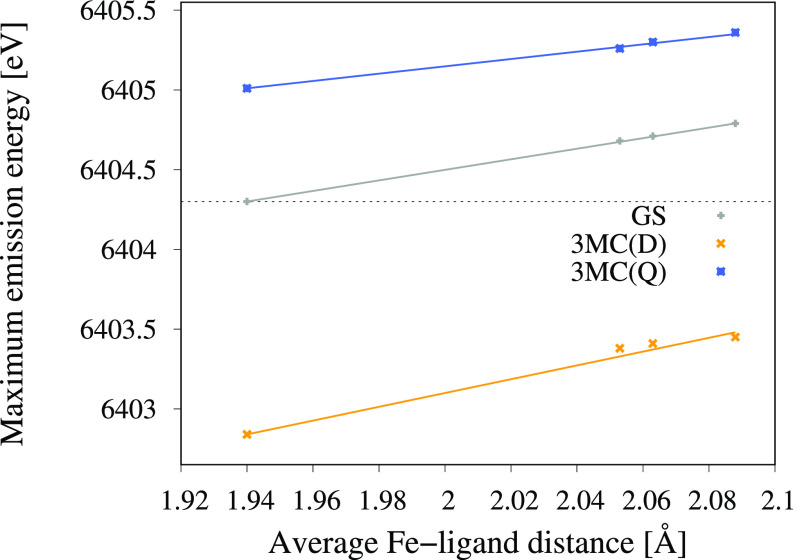
Energies of the K*α* emission maxima as a function of the Fe-ligand distance for the GS, ^3^MC(D), and ^3^MC(Q). The points are the data calculated at the four different nuclear geometries (GS minimum, ^3^MC short, ^3^MC minimum, and ^3^MC long), while the lines are linear fits through these data points. The horizontal dashed line indicates the energy of the GS emission maximum at the GS minimum geometry.

As shown previously by XSS measurements and wavepacket quantum dynamics simulations, upon photoexcitation, the system evolves on a ^3^MC excited state surface and oscillates along a Fe-ligand stretching mode.^23^ To model the sensitivity of XES to structural dynamics, we calculated emission spectra at several geometries, in addition to the GS minimum energy geometry, in the vicinity of the ^3^MC minimum around which the vibrational wavepacket oscillates (see Fig. 5). These calculations show that although the spectral shapes do not change much, there is a clear blueshift of the emission energies as the Fe-ligand distance increases. It is noted that the emission spectra at different ^3^MC geometries seem to cross at specific energies. However, these are not isosbestic points but are rather due to the relatively small shift in emission energy between the three curves, which makes different two-curve crossings appear close in energy. The energies of the K*α* emission maxima as a function of the Fe-ligand distance are shown in Figs. 6. The corresponding data for the higher-lying ^3^MC states are shown in Fig. S3 in the supplementary material.

Two possible explanations can be proposed to rationalize these results, either a redistribution of intensities between different final states or more simply an energy shift of the dominant transitions. A detailed analysis of the emission spectra at different geometries shows that, despite the contribution from a very large number of transitions, it is possible to identify the major contributions to intensity in the different parts of the spectra and to follow them as the geometry changes: all transitions show a clear blueshift with the increasing Fe-ligand distance, therefore validating the second of our possible explanations.

### B. Analyzing structural sensitivity of emission intensity

**FIG. 7.**
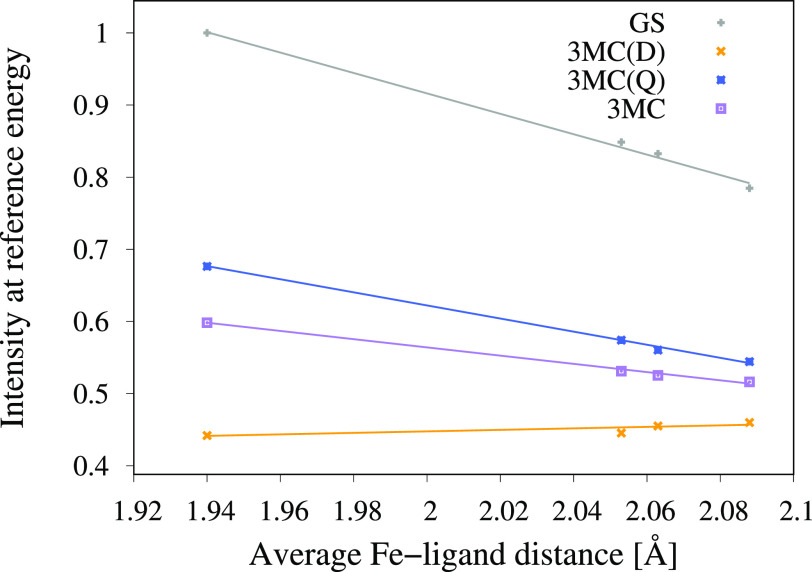
Emission intensities at the GS K*α* maximum energy, as a function of the Fe-ligand distance for the GS, ^3^MC(D), ^3^MC(Q), and ^3^MC. The latter is obtained from ^3^MC(Q) and ^3^MC(D) with a 4:2 ratio. The points are the data calculated at the four different nuclear geometries (GS minimum, ^3^MC short, ^3^MC minimum, and ^3^MC long), while the lines are linear fits through these data points.

Let us now look at the emission intensity at the energy of the GS K*α* maximum (6404.3 eV), which is experimentally observable.^23^ Figure 7 shows the intensities as a function of the Fe-ligand distance for the GS, ^3^MC(D), and ^3^MC(Q) states. Because of the energy shift of the GS emission spectrum as the Fe-ligand distance increases, the intensity of the GS spectrum decreases at the reference energy of 6404.3 eV. At the GS minimum geometry, the ^3^MC(Q) emission spectrum is blue shifted with respect to the GS emission spectrum. Since it gets further blue shifted as the Fe-ligand distance increases, the intensity of the ^3^MC(Q) spectrum decreases further at the reference energy. The ^3^MC(D) emission spectrum is however red shifted with respect to the GS emission spectrum at the GS minimum geometry. Since it gets blue shifted as the Fe-ligand distance increases, the intensity of the ^3^MC(D) spectrum actually increases at the reference energy. The increase is small due to the rather flat shape of the ^3^MC(D) spectra in this energy range.

The sensitivity of the emission intensity to structural changes shown in Fig. 7 can be expressed as

$$\begin{array}{c} \frac{\delta I(E)}{\delta r}=\frac{\delta I(E)}{\delta E_{\max}}\frac{\delta E_{\max}}{\delta r} \end{array}.$$ (1)

The first factor [$\delta I(E)/\delta E_{\max}$] is the change in intensity with respect to an energy shift of the emission curve. For small changes, this can be approximated as the negative value of the slope of the emission intensity at a given energy. This will be exemplified by the GS spectrum. At 6404.3 eV, the curve has a positive slope for the ^3^MC minimum structure (see Fig. 5). A shift of the emission curve to higher energies then leads to a decrease in the intensity [negative $\delta I(E)/\delta E_{\max}$]. Numerical values for different states, all obtained at the ^3^MC minimum energy structure, are given in Table I. The shape of the spectra of the intermediate states is very different, which in turn leads to large effects on the sensitivity. For the ^3^MC(Q) intermediate, the value is negative, while the ^3^MC(D) has a smaller positive value. As the former contributes more, this leads to an overall negative value for the ^3^MC emission.

**TABLE I.** Sensitivity of emission intensity to distance changes (δI/δr) and its two contributing factors. Two sensitivity values are shown, both the product of the two factors and the fitted value from Fig. 7.

Factor	GS	^3^MC(Q)	^3^MC(D)	^3^MC
$\delta I/\delta E_{\max}$ (eV^–1^)	−0.52	−0.33	+0.04	−0.21
$\delta E_{\max}/\delta r$ (eV ·Å^–1^)	3.3	2.3	4.4	3.0
δI/δr (Å^–1^) (Product)	−1.72	−0.76	+0.18	−0.63
δI/δr (Å^–1^) (Fit)	−1.51	−0.97	+0.11	−0.61

The second factor, $\delta E_{\max}/\delta r$, shows the sensitivity of the emission peak energies with regard to structural changes. As the shapes of the emission spectra are constant for oscillations around the ^3^MC minimum, this can be approximated by the slope of the emission maximum with respect to distance (see Fig. 6). These values are similar for different intermediate states, with all showing a positive slope between 2.3 and 4.4 eV ·Å^–1^ (see Table I). The origin of these peak shifts and whether similar shifts can be expected in general are analyzed in more detail below.

We note a good agreement between the global sensitivity extracted from Fig. 7 and the value obtained by taking the products of the two contributing factors (see Table I). This validates the qualitative interpretation of the sensitivity of emission intensity to distance changes in terms of simpler physical concepts.

### C. Origin of structural sensitivity

**FIG. 8.**
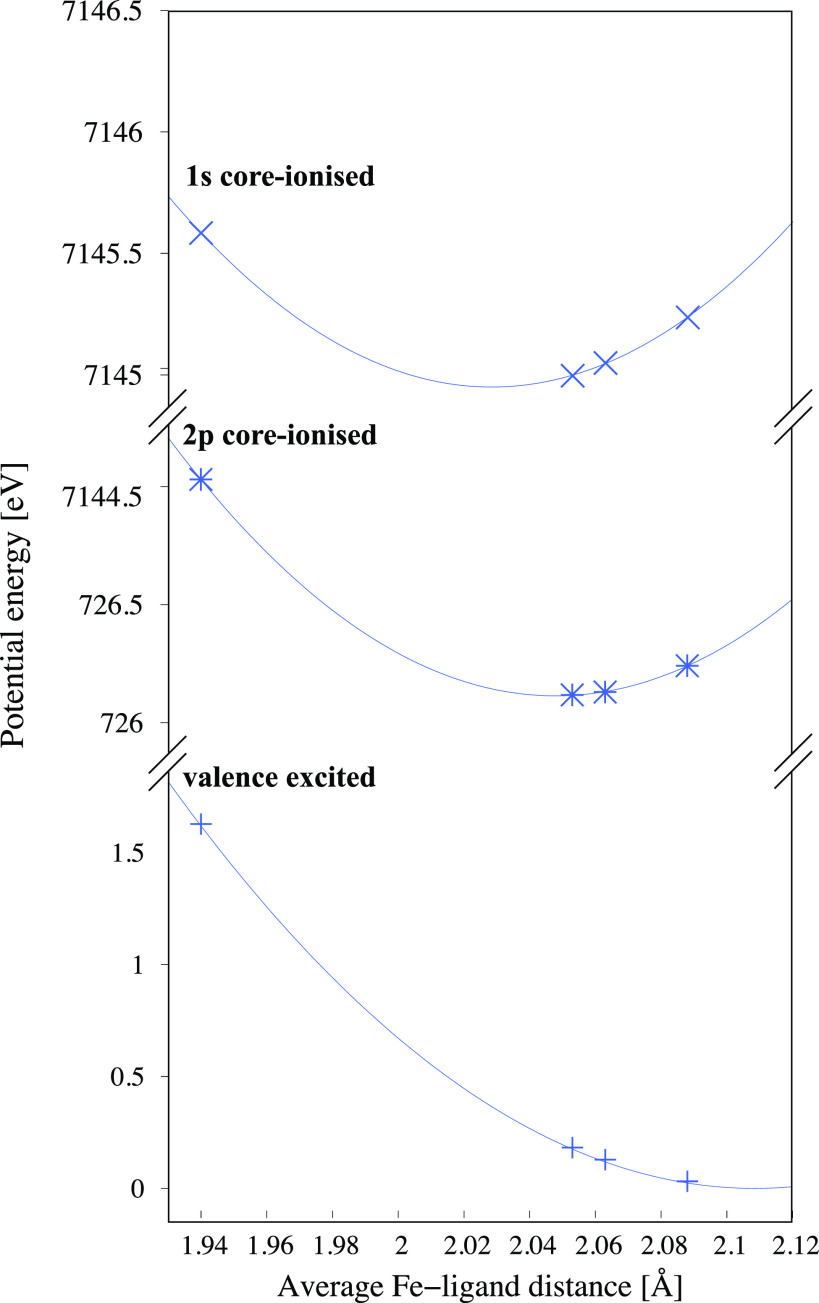
Potential energy curves of the ^3^MC valence state and the corresponding ^3^MC(Q) states with 1*s* and 2*p* holes along the GS minimum-^3^MC minimum coordinate.

From the previous analysis, it is clear that the maximum of the XES spectra of all involved states shifts to higher energy with the increasing metal–ligand bond distance. As the most important states involved in the emission process stay the same, at least for smaller geometry changes, following the energy of these states gives potential energy surfaces of the core-excited states (see Fig. 8). The results in this figure are similar, but not identical, to the ones in Ref. 23 because here we do the analysis at the level of spin-free states rather than spin–orbit coupled states. The former are used here because they facilitate comparisons between intermediate states with different spin-couplings. This additional transparency is helpful as the simulations do not explicitly treat the electron dynamics of the short-lived 1*s* core-excited state.

In the RASSCF calculations, the minimum of the ^3^MC valence state is at 2.11 Å, slightly higher than the DFT optimized structure used as the ^3^MC reference. In both 1*s* and 2*p* core-hole states, this minimum shifts to much shorter distances. The state with a 1*s* hole has a minimum 0.08 Å shorter than the valence ^3^MC state. Furthermore, there is also a clear difference in the potential energy surface (PES) depending on the location of the core hole, with the 1*s* hole giving the shortest distance by a margin of 0.019 Å. The curvatures of the PES of the core-hole states are very similar, with force constants (k) of 162.2 and 157.3 eV · Å^–2^ for 1*s* and 2*p* holes, respectively. The curvature for the valence state is smaller (114.1 eV · Å^–2^).

The increase in emission energy for longer distances during the coherent structural dynamics on the ^3^MC PES can be understood using the difference in the position of the energy minima ($\Delta r=r_{2p}-r_{1s}$). As the PESs are approximately quadratic near their minima, $E=\frac{1}{2}k{\left(r-r_{\min}\right)}^{2}+E_{\min}$, the energy difference between the 1*s* and 2*p* states becomes larger for longer distances even if the curvature would be the same. Assuming that the force constant is equal in the core-excited states $k_{ce}=k_{1s}=k_{2p}$ gives the following expressions for the sensitivity of the emission energy to the structure:

$$\begin{array}{c} E(r)=(E_{1s}-E_{2p})=k_{ce}r\Delta r+E_{\mathrm{constant}} \end{array},$$ (2)

$$\begin{array}{c} \frac{\delta E}{\delta r}=k_{ce}\Delta r \end{array}.$$ (3)

Using the values of *k_ce_* and Δr in the above equation, we get a value of δE/δr of 3.0 eV ·Å^−1^, in very good agreement with Fig. 6 and Table I. The sensitivity to the structure can thus be simply explained by the shift in the potential energy curves between states with different core holes. The effects of the core holes on metal–ligand bonding are therefore analyzed in detail below.

### D. Core-hole effects on metal–ligand bonding

**FIG. 9.**
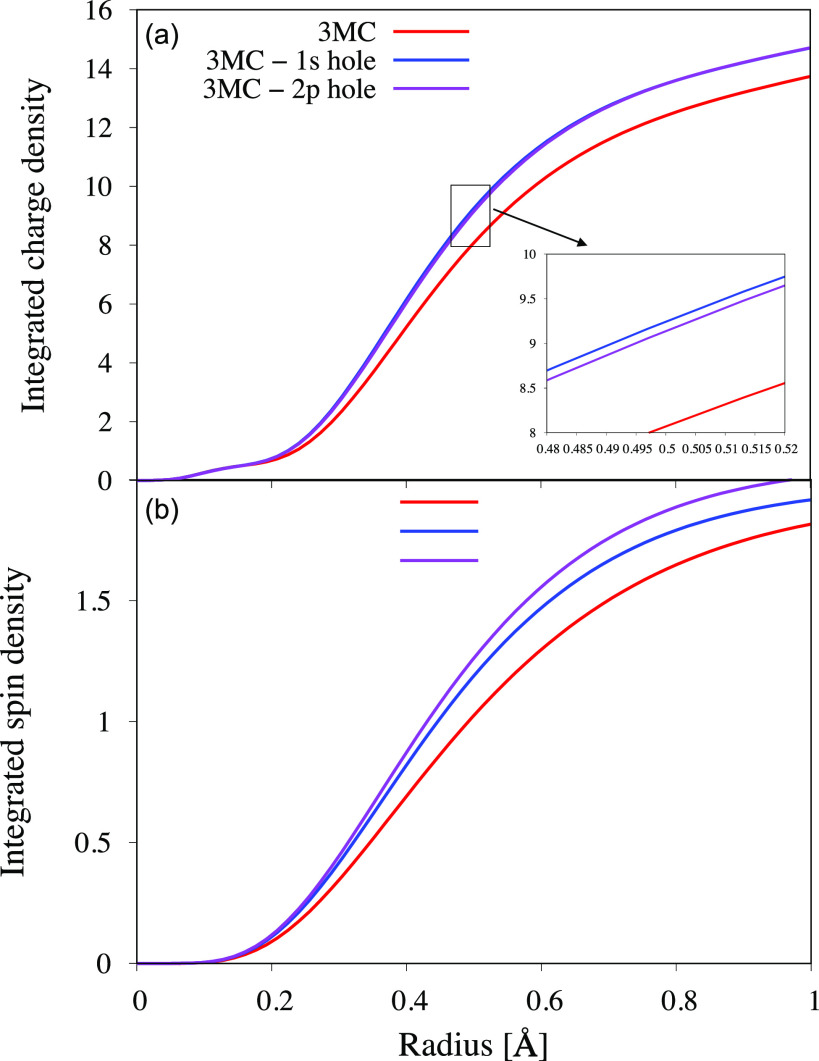
(a) Radial charge density and (b) radial spin density plots for the ^3^MC valence state and the corresponding states with 1*s* and 2*p* holes (calculated at the ^3^MC minimum geometry). The analysis does not include the core orbitals as the changes in orbital occupation would dominate the comparison between states.

The core-hole effects on the valence orbitals are analyzed using the radial charge and spin densities of the ^3^MC state with and without core holes [see Fig. 9(a)].^54^ As expected, the valence-electron distribution is shifted more toward the metal in the core-hole states. The effect on the charge and spin densities upon creation of a core hole is well understood in the molecular-orbital picture. Creation of a positive hole leads to a strong attractive force and a significant contraction of all metal orbitals. To illustrate these effects, orbital energies and pairwise interactions for the M-shell (3*s*, 3*p*, 3*d*) orbitals in an iron(II) atomic ion have been calculated using the atomic multiplet model (see Table II).^51^ Creation of a positive core hole lowers the orbital energy of all these orbitals. The increase in electron–nuclei interactions shows that the shift in orbital energies are not mainly due to a decrease in electron–electron repulsion but rather a contraction of the entire shell, just as seen in the charge-density plots.

**TABLE II.** Orbital energies and electron-nuclei interactions for an iron(II) atomic ion in the ground (GS) and core-excited (CE) states with 1*s* and 2*p* holes.

	Orbital energy (eV)			Nuclei interaction (eV)		
Orbital	GS	CE-1*s*	CE-2*p*	GS	CE-1*s*	CE-2*p*
3*s*	−134.3	−160.6	−158.5	−874.5	−895.8	−890.7
3*p*	−93.8	−120.3	−117.6	−819.3	−850.1	−842.4
3*d*	−34.5	−56.3	−56.9	−679.4	−738.6	−739.4

Furthermore, looking into the radial charge distribution, it is clear that the 1*s* hole leads to a more contracted valence shell. This is also expected from a pure atomic analysis. Since orbitals behave as *r^l^* close to the nucleus, the lower the *l* value of an orbital (s,p,d,f), the higher the density at the nuclei. Electron–electron interactions are especially large when two electrons are close to the nuclei, and therefore, bound electrons in general interact stronger with 1*s* than 2*p* particles.^55^ This effect is seen in Table III, where the pairwise interactions for all M-shell electrons are stronger with 1*s* compared to 2*p* electrons in all states. However, it should be noted that the difference is much smaller for 3*d* compared to 3*s* and 3*p* electrons.

**TABLE III.** Pairwise interactions (in eV) between M-shell and 1*s*/2*p* core electrons for an iron(II) atomic system in ground (GS) and core-excited (CE) states.

	GS		CE-1*s*		CE-2*p*	
Orbitals	1*s*	2*p*	1*s*	2*p*	1*s*	2*p*
3*s*	43.4	39.6	45.2	41.3	44.8	40.8
3*p*	42.3	38.1	44.9	40.4	44.3	39.8
3*d*	33.8	33.0	37.9	36.9	37.9	36.9

To further analyze the effects of the core holes on the molecular complex, the radial spin density was analyzed [see Fig. 9(b)]. As before, the core orbitals are not included in the calculations, so the analysis of the core-excited states reflects the non-zero spin in the $t_{2g}^{5}$ and $e_{g}^{1}$ sub-shells. The spin-density analysis thus gives equal weight to the metal 3*d*-dominated orbitals, while largely ignoring the other shells. There is a clear contraction of the density upon creation of the core hole, just as for the charge density. However, when looking at the spin density, it is further away from the metal in the 1*s* hole state than in the 2*p* state. The spin-density trend between the states thus deviates from the trend in the calculated PES minima in Fig. 8 and is in apparent contradiction with the stronger 1*s* core–hole interactions.

To reconcile these observations, we first look at the orbital energies in Table II in more detail. This shows that although 3*s* and 3*p* shells have significantly lower energies in the 1*s* core-hole state, the energy of the 3*d* shell actually increases. Using the electron–nuclei interaction as an indication of the orbital radius, these values show that 3*s* and 3*p* electrons are more contracted in the 1*s* state, while the 3*d* electrons are less contracted. The reason for this counter-intuitive effect is the significant spatial overlap of the M shells. The additional contraction of the 3*s* and 3*p* orbitals in the 1*s* core-hole state leads to an increased shielding of the nuclear charge for the 3*d* orbitals. This shielding leads to an expansion of the 3*d* orbitals, which dominates over the contraction effect from the slightly stronger 1*s* core-hole attraction. The radial charge and spin densities are thus fully consistent with a simple atomic analysis.

**FIG. 10.**
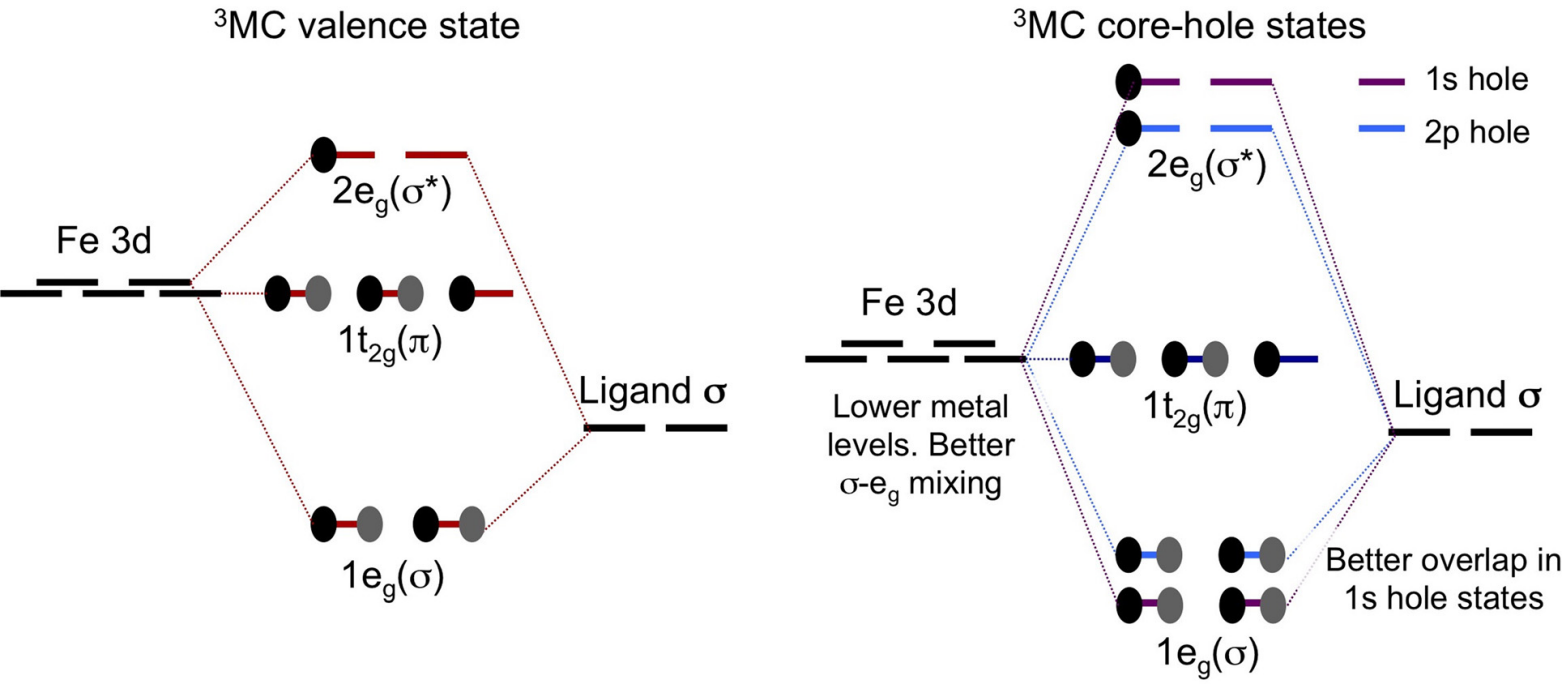
Two-configuration model explaining increased covalency in core-hole states and differences between 1*s* and 2*p* holes.

Still, the relation between electron density and the relative positions of the PES minima needs to be explained. For this purpose, we have used a simple two-configuration model. As the complex is dominated by *σ*-donor bonding from low-lying filled ligand orbitals, lowering the metal levels upon creation of a core hole decreases the energy difference between metal and ligand configurations (see Fig. 10). This improved match gives stronger mixing and a more covalent bond, as seen from the increased covalency of the *σ* and *e_g_* orbitals (see Table IV). Furthermore, contraction of the entire valence shell leads to a smaller ionic radius, which can give a shorter bond without directly affecting its character. The relative magnitude of these two contributions to the observed bond-length contraction of 0.06–0.08 Å upon core-hole creation is difficult to separate.

**TABLE IV.** Orbital covalency analysis for the metal–ligand orbitals in different states.

	GS geometry			^3^MC geometry		
State	*σ*^a^	$t_{2}g$	*e_g_*^a^	*Σ*^a^	$t_{2}g$	*e_g_*^a^
^3^MC	18.8	92.7	77.3	9.2	91.7	89.9
^3^MC 1*s* hole	22.4	93.1	73.3	15.7	95.7	79.3
^3^MC 2*p* hole	21.8	94.8	72.3	15.3	95.7	79.2

^a^ Data from Ref. 23.

When comparing the two core-hole states, the shorter bond distances in the 1*s* core-hole states do not seem to match with the observation that the atomic 3*d* orbitals expand in this state. Bond-length shortening must therefore come from significant contraction of the non-bonding 3*s* and 3*p* electrons. One possibility is that this contraction reduces the Coulomb repulsion with the ligand electrons, which leads to better overlap between metal 3*d* and ligand levels and consequently larger off diagonal elements in the two-configuration model (see Fig. 10). The 1*s* core-hole state also has slightly larger *σ* and *e_g_* covalencies, more pronounced at shorter distances (see Table IV). A second possibility is a decrease in the atomic radius, which would give a shorter bond without directly affecting the character of the bond. Combined, these two effects lead to the 0.019 Å additional contraction in the 1*s* compared to the 2*p* core-hole state.

The general idea that removing non-bonding electrons can lead to increased *σ*-donation in metal-ligand bonding is supported by femtosecond resonant inelastic x-ray spectroscopy of the ligand-to-metal charge-transfer excited state of ferrocyanide.^56^ The significant effects of the 3*s* and 3*p* orbitals on bonding are consistent with the importance of 3*s*-3*p* hybridization to minimize ligand repulsion and increase bonding^57^ and the role of explicit 3*s* and 3*p* correlation in high-accuracy modeling of coordination complexes.^58^

### E. Connection to the chemical shift in K*α* emission

The importance of 3*s* and 3*p* orbitals in K*α* XES of transition metals is already well established from analyses of the chemical shift. For light elements, oxidation leads to a positive shift in the K*α* emission energies. The removal of a valence electron decreases the electron density near the nucleus, and as the interactions are more favorable with a 1*s* compared to a 2*p* hole, this leads to higher energy for the 2p→1s transition.^55,59^ However, transition metal compounds typically show negative shifts upon oxidation.^60–62^ This can be explained by the presence of two counteracting effects, a first-order effect coming from the direct loss of 3*d* electron density and a second-order effect coming from the compression of all metal orbitals, especially 3*s* and 3*p*, upon the loss of the 3*d* electron. As 3*s* and 3*p* electrons interact more strongly with the core holes than the 3*d* electrons, their negative contribution dominates the K*α* shift for 3*d* transition metals. The experimental observation of a negative chemical shift for transition metals shows that 3*s* and 3*p* electrons can have dominant influences on K*α* XES even if they are not directly involved in redox activity or chemical bonding.

### F. Predicting sensitivity of K*α* XES to structural dynamics

**FIG. 11.**
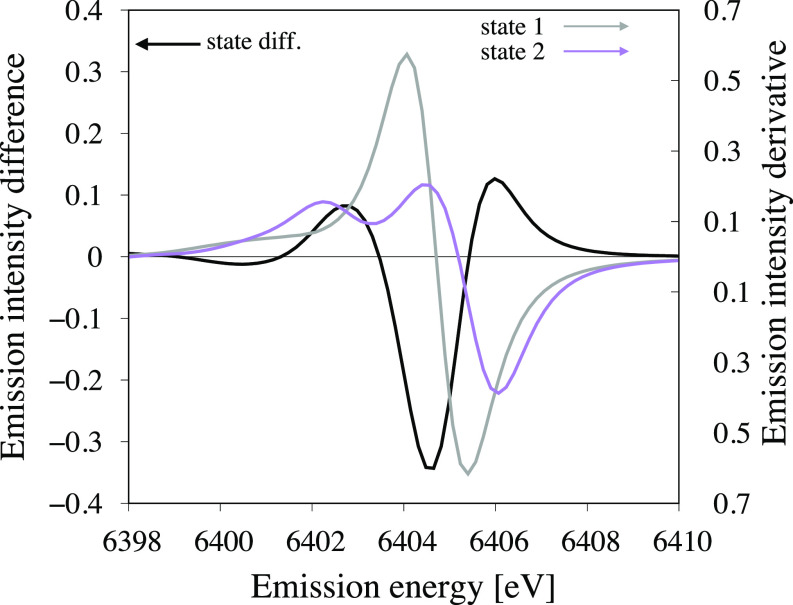
Sensitivity to electron and structural dynamics in two states as a function of the emission energy. Sensitivity to electron dynamics is calculated as the difference in intensity between the two electronic states (black curve, left *y*-axis). Sensitivity to structural dynamics is calculated as the emission intensity variation for two states, using a vibrational oscillation of 0.025 Å (gray and purple curves, right *y*-axis). All calculations are performed at the ^3^MC minimum energy structure.

For future applications of XES to study structural dynamics, it is important to predict conditions that will show a large sensitivity to such changes. To detect the wavepacket dynamics, the emission intensity must then be sensitive to the structural variations (δI/δr). This sensitivity can be predicted from the XES spectra for the relevant states. To be completely general, we call these states 1 and 2, although in the current example these are the GS and ^3^MC states, respectively. Figure 11 (gray and purple curves, right *y*-axis) shows the changes in emission intensity (ΔI) as a function of the emission energy for the two different states, calculated using a maximum oscillation of the nuclear wavepacket of 0.025 Å.^23^ A high sensitivity to structural dynamics in state 2 (^3^MC) is found at 6404.5 eV, which is close to the value chosen in the experiment (6404.3 eV). Sensitivity is actually even larger, up to 4% of the maximum intensity, when measuring on the high-energy side of the XES spectrum, around 6406.0 eV.

Tuning the emission spectrometer also makes it possible to switch between observing electronic and structural dynamics. At certain energies, the emission intensities of the two states are equal, making the XES signal independent of the electronic state [see Fig. 11 (black curve, left *y*-axis)]. At the same time, the signal should be off resonance for at least one state, in this example state 1, leading to sensitivity to geometric structure changes. Further increasing the emission energy again leads to sensitivity to electron dynamics.

**FIG. 12.**
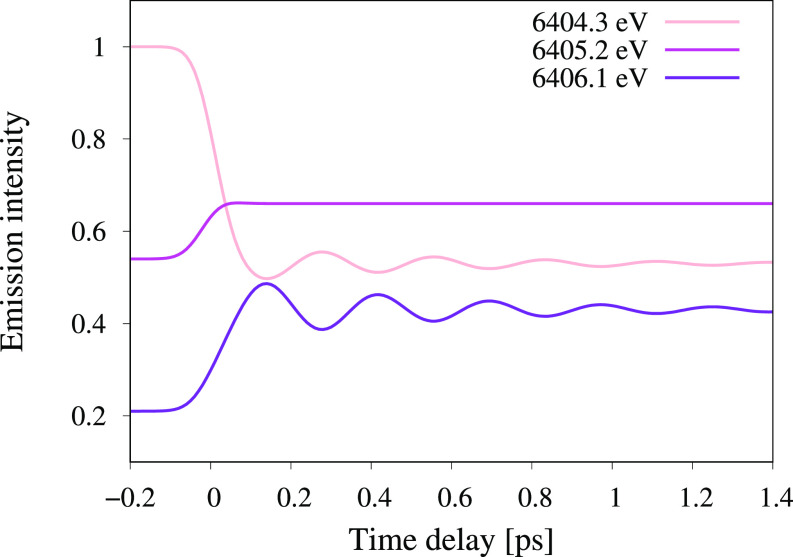
Predicted time-dependent XES intensities for a two-state system at different spectrometer emission energies. Bond distances as a function of time are taken from a previous wavepacket dynamics simulation.^23^

To illustrate how the emission energy affects the experimental observable, time-dependent XES spectra have been simulated for three different emission energies (see Fig. 12). The emission energies are chosen based on the results in Fig. 11 and represent situations ranging from minimum to maximum sensitivity to structural dynamics. For simplicity, the simulations assume a two-state system with a 100% quantum yield and an infinite lifetime of the final state. However, as the magnitude of the oscillations is proportional to changes in the bond distance, the simulations require time-dependent bond distances. Here, this information is taken from previous wavepacket dynamics simulations of [Fe^*II*^(bmip)_2_]^2+^, including the additional Gaussian broadening of 100 fs (FWHM) to account for experimental time-resolution.^23^

The first example represents the commonly chosen experimental condition where data are collected at the emission maximum of state 1 (GS at 6404.3 eV). The initial intensity is then unity and drops to below 0.6 as the system transfers to state 2 (^3^MC). Note that the intensity difference between the states is not exactly the same as in Figs. 4 and Fig. 11 because the current modeling also includes changes in geometry. At this emission energy, there is significant structural sensitivity, with the largest oscillations of around ±2%. These oscillations then decrease with time as the wavepacket dephases. If the emission energy instead moves to the maximum emission intensity of state 2 (^3^MC at 6405.2 eV), the transfer from state 1 to state 2 increases the intensity, but there is no structural sensitivity as the derivative of the intensity with respect to energy is zero at the maximum. Finally, increasing the emission energy to the point of maximum sensitivity of state 2 (6406.1 eV) leads to low total intensities but large oscillations, around ±4%. Together, these three examples show how the emission energy can be used to modulate the structural sensitivity of the experiment. This in turn opens up for separation of electronic and geometric degrees of freedom in XES studies of ultrafast dynamics.

### G. Other systems and detection methods

These time-dependent XES simulations using data from [Fe^II^(bmip)_2_]^2+^ provide a framework for discussing if structural sensitivity of K*α* XES is important for a wider range of systems. The present theoretical analysis has identified the following factors that need to be considered: (i) large differences in metal–ligand equilibrium distances between 1*s* and 2*p* core-hole states (large Δr, see Eq. 3), (ii) strong metal–ligand bonds in the core-excited states (large *k_ce_*), and (iii) emission energy properly tuned to detect the oscillations.

The first factor, Δr of the core-excited states, is predicted to be general because its main origin is an atomic effect. It should therefore not vary significantly between systems or electronic states. For [Fe^II^(bmip)_2_]^2+^, the effect is similar for both GS and ^3^MC electronic states, even if these states have very different metal–ligand bonding. The dependence of the oscillations on the force constant means that the systems with strong metal–ligand bonds, such as those from the strong *σ* bonding in the carbenes, are more sensitive. Still, as the effect is linear, significant oscillations should appear also in other systems. These values can be obtained from DFT or time-dependent DFT simulations of the targeted excited states and compared to the force constant of 2000 Nm^−1^ extracted from these simulations. Finally, the selected emission energy is possibly the most sensitive variable. To design new experiments targeting electronic and structural dynamics in excited states, this emission energy can be chosen based on theoretical predictions as in Fig. 11. Although the theoretical analysis here indicates the possibility of fluctuations up to 4% of the maximum emission intensity, the generality of these findings will have to be tested through further theoretical and experimental work on coherently excited states.

In addition to the predicted structural sensitivity in K*α* XES, an important question is whether effects can also be expected in K*β* XES. For [Fe^*II*^(bmip)_2_]^2+^, there are indications that oscillations occur also in the K*β* signal, although the low signal-to-noise ratio prevents a definitive confirmation.^23^ Here, we can only observe that the factors outlined for structural sensitivity of K*α* XES cannot be directly transferred to K*β* XES. First, the radial distribution of the 3*p* hole overlaps more directly with the valence orbitals, which can lead to effects on chemical bonding beyond the predominantly atomic effects of the deeper core holes. The shifts in the PES surfaces of different core hole states can therefore be more dependent on the metal-ligand bonding characteristics. Second, the K*β* and K*α* emission data were collected in different experimental setups. As mentioned above, K*α* XES was detected using a Johann spectrometer, which is sensitive to a narrow emission energy range. The K*β* signal is collected using a dispersive von Hamos spectrometer. The experimental signal-to-noise ratio does not allow the extraction of intensity trends at specific energies, but weak oscillations appear by integrating over the full spectral range.^23^ Due to the lower K*β* fluorescence yield, the experimental signal-to-noise ratio in that particular experiment was about 10 times lower compared to K*α* XES. If the main effect is an energy shift, the integrated signal would only be sensitive to the difference in intensity changes at the two ends of the emission energy interval, where the signal is already weak. For [Fe^*II*^(bmip)_2_]^2+^, it is thus possible that the K*β* oscillations reflect changes in spectral intensity instead of an energy shift. To understand the sensitivity of K*β* XES to structural dynamics requires further simulations of that particular process.

## IV. CONCLUSIONS

The sensitivity of XES emission to excited-state structural dynamics in an iron photosensitizer is due to the shift of the minimum metal-ligand bond distance between 1*s* and 2*p* core-hole states. A key effect comes from contraction of the non-bonding 3*s* and 3*p* orbitals in 1*s* core-hole states, which decreases electron-electron repulsion and increases overlap in metal–ligand bonding. The difference between the 1*s* and 2*p* core hole potentials explains trends in radial charge densities, the shapes of the PES of the core-hole states, and the subsequent shift in the emission energy with the metal–ligand distance. The explanation is also consistent with the negative chemical shift observed in transition metal complexes. The atomic origin of the process predicted here suggests that the structural sensitivity of K*α* emission is a general effect and should be observed in other molecular systems. Furthermore, the sensitivity to electronic and structural dynamics in different states can be tuned by changing the emission energy, making it possible to selectively study either oscillations between electronic states or structural changes in a specific state. The results show how modeling can be used to predict sensitivity as a function of emission energy and thus help to improve experiments designed to follow ultrafast excited state dynamics.

## SUPPLEMENTARY MATERIAL

See the supplementary material for the effects of dynamic correlation on the emission spectrum, the description of other ^3^MC intermediate states, their K*α* emission spectra, the energies of their emission maximum, and Cartesian coordinates of the different nuclear geometries used.

## Data Availability

The data that support the findings of this study are available from the corresponding author upon reasonable request.

## References

[1] H. B. Gray and A. W. Maverick , “ Solar chemistry of metal complexes,” Science 214, 1201–1205 (1981). 10.1126/science.214.4526.1201 17789279

[2] C. S. Ponseca , P. Chábera , J. Uhlig , P. Persson , and V. Sundström , “ Ultrafast electron dynamics in solar energy conversion,” Chem. Rev. 117, 10940–11024 (2017). 10.1021/acs.chemrev.6b00807 28805062

[3] Y. Liu , P. Persson , V. Sundström , and K. Wärnmark , “ Fe N-heterocyclic carbene complexes as promising photosensitizers,” Acc. Chem. Res. 49, 1477–1485 (2016). 10.1021/acs.accounts.6b00186 27455191

[4] M. Chergui and E. Collet , “ Photoinduced structural dynamics of molecular systems mapped by time-resolved x-ray methods,” Chem. Rev. 117, 11025–11065 (2017). 10.1021/acs.chemrev.6b00831 28692268

[5] M. Chergui , “ Ultrafast photophysics of transition metal complexes,” Acc. Chem. Res. 48, 801–808 (2015). 10.1021/ar500358q 25646968

[6] W. Zhang , R. Alonso-Mori , U. Bergmann , C. Bressler , M. Chollet , A. Galler , W. Gawelda , R. G. Hadt , R. W. Hartsock , T. Kroll , K. S. Kjær , K. Kubiček , H. T. Lemke , H. W. Liang , D. a Meyer , M. M. Nielsen , C. Purser , J. S. Robinson , E. I. Solomon , Z. Sun , D. Sokaras , T. B. van Driel , G. Vankó , T.-C. Weng , D. Zhu , and K. J. Gaffney , “ Tracking excited-state charge and spin dynamics in iron coordination complexes,” Nature 509, 345–348 (2014). 10.1038/nature13252 24805234 PMC5668134

[7] S. E. Canton , K. S. Kjær , G. Vankó , T. B. van Driel , S-I Adachi , A. Bordage , C. Bressler , P. Chabera , M. Christensen , A. O. Dohn , A. Galler , W. Gawelda , D. Gosztola , K. Haldrup , T. Harlang , Y. Liu , K. B. Møller , Z. Németh , S. Nozawa , M. Pápai , T. Sato , T. Sato , K. Suarez-Alcantara , T. Togashi , K. Tono , J. Uhlig , D. A. Vithanage , K. Wärnmark , M. Yabashi , J. Zhang , V. Sundström , and M. M. Nielsen , “ Visualizing the non-equilibrium dynamics of photoinduced intramolecular electron transfer with femtosecond x-ray pulses,” Nat. Commun. 6, 6359 (2015). 10.1038/ncomms7359 25727920 PMC4366532

[8] M. W. Mara , R. G. Hadt , M. E. Reinhard , T. Kroll , H. Lim , R. W. Hartsock , R. Alonso-Mori , M. Chollet , J. M. Glownia , S. Nelson , D. Sokaras , K. Kunnus , K. O. Hodgson , B. Hedman , U. Bergmann , K. J. Gaffney , and E. I. Solomon , “ Metalloprotein entatic control of ligand-metal bonds quantified by ultrafast x-ray spectroscopy,” Science 356, 1276–1280 (2017). 10.1126/science.aam6203 28642436 PMC5706643

[9] K. S. Kjær , K. Kunnus , T. C. B. Harlang , T. B. Van Driel , K. Ledbetter , R. W. Hartsock , M. E. Reinhard , S. Koroidov , L. Li , M. G. Laursen , E. Biasin , F. B. Hansen , P. Vester , M. Christensen , K. Haldrup , M. M. Nielsen , P. Chabera , Y. Liu , H. Tatsuno , C. Timm , J. Uhlig , V. Sundstöm , Z. Németh , D. S. Szemes , É. Bajnóczi , G. Vankó , R. Alonso-Mori , J. M. Glownia , S. Nelson , M. Sikorski , D. Sokaras , H. T. Lemke , S. E. Canton , K. Wärnmark , P. Persson , A. A. Cordones , and K. J. Gaffney , “ Solvent control of charge transfer excited state relaxation pathways in, [Fe(2,2–bipyridine)(CN)_4_]^2^,” Phys. Chem. Chem. Phys. 20, 4238–4249 (2018). 10.1039/C7CP07838B 29364300

[10] K. S. Kjær , T. B. Van Driel , T. C. B. Harlang , K. Kunnus , E. Biasin , K. Ledbetter , R. W. Hartsock , M. E. Reinhard , S. Koroidov , L. Li , M. G. Laursen , F. B. Hansen , P. Vester , M. Christensen , K. Haldrup , M. M. Nielsen , A. O. Dohn , M. I. Pápai , K. B. Møller , P. Chabera , Y. Liu , H. Tatsuno , C. Timm , M. Jarenmark , J. Uhlig , V. Sundstöm , K. Wärnmark , P. Persson , Z. Németh , D. S. Szemes , É. Bajnóczi , G. Vankó , R. Alonso-Mori , J. M. Glownia , S. Nelson , M. Sikorski , D. Sokaras , S. E. Canton , H. T. Lemke , and K. J. Gaffney , “ Finding intersections between electronic excited state potential energy surfaces with simultaneous ultrafast X-ray scattering and spectroscopy,” Chem. Sci. 10, 5749–5760 (2019). 10.1039/C8SC04023K 31293761 PMC6568243

[11] H. Tatsuno , K. S. Kjær , K. Kunnus , T. C. B. Harlang , C. Timm , M. Guo , P. Chàbera , L. A. Fredin , R. W. Hartsock , M. E. Reinhard , S. Koroidov , L. Li , A. A. Cordones , O. Gordivska , O. Prakash , Y. Liu , M. G. Laursen , E. Biasin , F. B. Hansen , P. Vester , M. Christensen , K. Haldrup , Z. Németh , D. Sárosiné Szemes , É. Bajnóczi , G. Vankó , T. B. Van Driel , R. Alonso-Mori , J. M. Glownia , S. Nelson , M. Sikorski , H. T. Lemke , D. Sokaras , S. E. Canton , A. O. Dohn , K. B. Møller , M. M. Nielsen , K. J. Gaffney , K. Wärnmark , V. Sundström , P. Persson , and J. Uhlig , “ Hot branching dynamics in a light-harvesting iron carbene complex revealed by ultrafast x-ray emission spectroscopy,” Angew. Chem., Int. Ed. 59, 364–372 (2020). 10.1002/anie.201908065 31602726

[12] G. Peng , F. Degroot , K. Hämäläinen , J. Moore , X. Wang , M. Grush , J. Hastings , D. Siddons , and W. Armstrong , “ High-resolution manganese x-ray fluorescence spectroscopy. oxidation-state and spin-state sensitivity,” J. Am. Chem. Soc. 116, 2914–2920 (1994). 10.1021/ja00086a024

[13] P. Glatzel and U. Bergmann , “ High resolution 1s core hole x-ray spectroscopy in 3d transition metal complexes-electronic and structural information,” Coord. Chem. Rev. 249, 65–95 (2005). 10.1016/j.ccr.2004.04.011

[14] G. Vankó , A. Bordage , P. Glatzel , E. Gallo , M. Rovezzi , W. Gawelda , A. Galler , C. Bressler , G. Doumy , A. M. March , E. P. Kanter , L. Young , S. H. Southworth , S. E. Canton , J. Uhlig , G. Smolentsev , V. Sundström , K. Haldrup , T. B. van Driel , M. M. Nielsen , K. S. Kjaer , and H. T. Lemke , “ Spin-state studies with XES and RIXS: From static to ultrafast,” J. Electron Spectrosc. Relat. Phenom. 188, 166–171 (2013). 10.1016/j.elspec.2012.09.012

[15] H. Ihee , M. Lorenc , T. K. Kim , Q. Y. Kong , M. Cammarata , J. H. Lee , S. Bratos , and M. Wulff , “ Ultrafast x-ray diffraction of transient molecular structures in solution,” Science 309, 1223–1227 (2005). 10.1126/science.1114782 16020695

[16] H. Ihee , “ Visualizing solution-phase reaction dynamics with time-resolved x-ray liquidography,” Acc. Chem. Res. 42, 356–366 (2009). 10.1021/ar800168v 19117426

[17] K. H. Kim , J. G. Kim , S. Nozawa , T. Sato , K. Y. Oang , T. W. Kim , H. Ki , J. Jo , S. Park , C. Song , T. Sato , K. Ogawa , T. Togashi , K. Tono , M. Yabashi , T. Ishikawa , J. Kim , R. Ryoo , J. Kim , H. Ihee , and S-I Adachi , “ Direct observation of bond formation in solution with femtosecond x-ray scattering,” Nature 518, 385–389 (2015). 10.1038/nature14163 25693570

[18] D. Arnlund , L. C. Johansson , C. Wickstrand , A. Barty , G. J. Williams , E. Malmerberg , J. Davidsson , D. Milathianaki , D. P. DePonte , R. L. Shoeman , D. Wang , D. James , G. Katona , S. Westenhoff , T. A. White , A. Aquila , S. Bari , P. Berntsen , M. Bogan , T. B. van Driel , R. B. Doak , K. S. Kjær , M. Frank , R. Fromme , I. Grotjohann , R. Henning , M. S. Hunter , R. A. Kirian , I. Kosheleva , C. Kupitz , M. Liang , A. V. Martin , M. M. Nielsen , M. Messerschmidt , M. M. Seibert , J. Sjöhamn , F. Stellato , U. Weierstall , N. A. Zatsepin , J. C. H. Spence , P. Fromme , I. Schlichting , S. Boutet , G. Groenhof , H. N. Chapman , and R. Neutze , “ Visualizing a protein quake with time-resolved x-ray scattering at a free-electron laser,” Nat. Methods 11, 923–926 (2014). 10.1038/nmeth.3067 25108686 PMC4149589

[19] T. B. van Driel , K. S. Kjær , R. W. Hartsock , A. O. Dohn , T. Harlang , M. Chollet , M. Christensen , W. Gawelda , N. E. Henriksen , J. G. Kim , K. Haldrup , K. H. Kim , H. Ihee , J. Kim , H. Lemke , Z. Sun , V. Sundström , W. Zhang , D. Zhu , K. B. Møller , M. M. Nielsen , and K. J. Gaffney , “ Atomistic characterization of the active-site solvation dynamics of a model photocatalyst,” Nat. Commun. 7, 13678 (2016). 10.1038/ncomms13678 27892472 PMC5133712

[20] E. Biasin , T. B. van Driel , K. S. Kjær , A. O. Dohn , M. Christensen , T. Harlang , P. Chabera , Y. Liu , J. Uhlig , M. Pápai , Z. Németh , R. Hartsock , W. Liang , J. Zhang , R. Alonso-Mori , M. Chollet , J. M. Glownia , S. Nelson , D. Sokaras , T. A. Assefa , A. Britz , A. Galler , W. Gawelda , C. Bressler , K. J. Gaffney , H. T. Lemke , K. B. Møller , M. M. Nielsen , V. Sundström , G. Vankó , K. Wärnmark , S. E. Canton , and K. Haldrup , “ Femtosecond x-ray scattering study of ultrafast photoinduced structural dynamics in solvated, [Co(terpy)_2_]^2+^,” Phys. Rev. Lett. 117, 013002 (2016). 10.1103/PhysRevLett.117.013002 27419566

[21] D. Leshchev , T. C. B. Harlang , L. A. Fredin , D. Khakhulin , Y. Liu , E. Biasin , M. G. Laursen , G. E. Newby , K. Haldrup , M. M. Nielsen , K. Wärnmark , V. Sundström , P. Persson , K. S. Kjær , and M. Wulff , “ Tracking the picosecond deactivation dynamics of a photoexcited iron carbene complex by time-resolved x-ray scattering,” Chem. Sci. 9, 405–414 (2018). 10.1039/C7SC02815F 29629111 PMC5868308

[22] K. Haldrup , G. Levi , E. Biasin , P. Vester , M. G. Laursen , F. Beyer , K. S. Kjær , T. Brandt van Driel , T. Harlang , A. O. Dohn , R. J. Hartsock , S. Nelson , J. M. Glownia , H. T. Lemke , M. Christensen , K. J. Gaffney , N. E. Henriksen , K. B. Møller , and M. M. Nielsen , “ Ultrafast x-ray scattering measurements of coherent structural dynamics on the ground-state potential energy surface of a diplatinum molecule,” Phys. Rev. Lett. 122, 063001 (2019). 10.1103/PhysRevLett.122.063001 30822093

[23] K. Kunnus , M. Vacher , T. C. B. Harlang , K. S. Kjær , K. Haldrup , E. Biasin , T. B. van Driel , M. Pápai , P. Chabera , Y. Liu , H. Tatsuno , C. Timm , E. Källman , M. Delcey , R. W. Hartsock , M. E. Reinhard , S. Koroidov , M. G. Laursen , F. B. Hansen , P. Vester , M. Christensen , L. Sandberg , Z. Németh , D. S. Szemes , É. Bajnóczi , R. Alonso-Mori , J. M. Glownia , S. Nelson , M. Sikorski , D. Sokaras , H. T. Lemke , S. E. Canton , K. B. Møller , M. M. Nielsen , G. Vankó , K. Wärnmark , V. Sundström , P. Persson , M. Lundberg , J. Uhlig , and K. J. Gaffney , “ Vibrational wavepacket dynamics in Fe carbene photosensitizer determined with femtosecond x-ray emission and scattering,” Nat. Commun. 11, 634 (2020). 10.1038/s41467-020-14468-w 32005815 PMC6994595

[24] P. J. Chirik , “ Iron-and cobalt-catalyzed alkene hydrogenation: Catalysis with both redox-active and strong field ligands,” Acc. Chem. Res. 48, 1687–1695 (2015). 10.1021/acs.accounts.5b00134 26042837

[25] O. S. Wenger , “ Is iron the new ruthenium?,” Chem.–A Eur. J. 25, 6043–6052 (2019). 10.1002/chem.201806148 30615242

[26] J. K. McCusker , “ Electronic structure in the transition metal block and its implications for light harvesting,” Science 363, 484–488 (2019). 10.1126/science.aav9104 30705184

[27] Y. Liu , T. Harlang , S. E. Canton , P. Chábera , K. Suárez-Alcántara , A. Fleckhaus , D. A. Vithanage , E. Göransson , A. Corani , R. Lomoth , V. Sundström , and K. Wärnmark , “ Towards longer-lived metal-to-ligand charge transfer states of iron (ii) complexes: An N-heterocyclic carbene approach,” Chem. Commun. 49, 6412–6414 (2013). 10.1039/c3cc43833c 23752944

[28] T. C. B. Harlang , Y. Liu , O. Gordivska , L. A. Fredin , C. S. Ponseca , P. Huang , P. Chábera , K. S. Kjaer , H. Mateos , J. Uhlig , R. Lomoth , R. Wallenberg , S. Styring , P. Persson , V. Sundström , and K. Wärnmark , “ Iron sensitizer converts light to electrons with 92% yield,” Nat. Chem. 7, 883–889 (2015). 10.1038/nchem.2365 26492008

[29] J. Olsen , B. O. Roos , P. Jo/rgensen , and H. J. A. Jensen , “ Determinant based configuration interaction algorithms for complete and restricted configuration interaction spaces,” J. Chem. Phys. 89, 2185–2192 (1988). 10.1063/1.455063

[30] P. Å. Malmqvist , A. Rendell , and B. O. Roos , “ The restricted active space self-consistent-field method, implemented with a split graph unitary group approach,” J. Phys. Chem. 94, 5477–5482 (1990). 10.1021/j100377a011

[31] I. Josefsson , K. Kunnus , S. Schreck , A. Föhlisch , F. de Groot , P. Wernet , and M. Odelius , “ Ab initio calculations of x-ray spectra: Atomic multiplet and molecular orbital effects in a multiconfigurational SCF approach to the L-edge spectra of transition metal complexes,” J. Phys. Chem. Lett. 3, 3565–3570 (2012). 10.1021/jz301479j 26290989

[32] S. I. Bokarev , M. Dantz , E. Suljoti , O. Kühn , and E. F. Aziz , “ State-dependent electron delocalization dynamics at the solute-solvent interface: Soft-x-ray absorption spectroscopy and *ab initio* calculations,” Phys. Rev. Lett. 111, 083002–083007 (2013). 10.1103/PhysRevLett.111.083002 24010434

[33] R. V. Pinjari , M. G. Delcey , M. Guo , M. Odelius , and M. Lundberg , “ Restricted active space calculations of L-edge x-ray absorption spectra: From molecular orbitals to multiplet states,” J. Chem. Phys. 141, 124116 (2014). 10.1063/1.4896373 25273421

[34] M. Guo , L. K. Sørensen , M. G. Delcey , R. V. Pinjari , and M. Lundberg , “ Simulations of iron K pre-edge x-ray absorption spectra using the restricted active space method,” Phys. Chem. Chem. Phys. 18, 3250–3259 (2016). 10.1039/C5CP07487H 26742851

[35] M. G. Delcey , L. K. Sørensen , M. Vacher , R. C. Couto , and M. Lundberg , “ Efficient calculations of a large number of highly excited states for multiconfigurational wavefunctions,” J. Comput. Chem. 40, 1789–1799 (2019). 10.1002/jcc.25832 30938847

[36] A. Tanaka and T. Jo , “ Resonant 3d, 3p, and 3s photoemission in transition metal oxides predicted at 2p threshold,” J. Phys. Soc. Jpn. 63, 2788–2807 (1994). 10.1143/JPSJ.63.2788

[37] F. de Groot , “ Multiplet effects in x-ray spectroscopy,” Coordin. Chem. Rev. 249, 31–63 (2005). 10.1016/j.ccr.2004.03.018

[38] L. A. Fredin , M. Pápai , E. Rozsályi , G. Vankó , K. Wärnmark , V. Sundström , and P. Persson , “ Exceptional excited-state lifetime of an iron(ii)-N-heterocyclic carbene complex explained,” J. Phys. Chem. Lett. 5, 2066–2071 (2014). 10.1021/jz500829w 26270494

[39] I. Fdez. Galván , M. Vacher , A. Alavi , C. Angeli , F. Aquilante , J. Autschbach , J. J. Bao , S. I. Bokarev , N. A. Bogdanov , R. K. Carlson , L. F. Chibotaru , J. Creutzberg , N. Dattani , M. G. Delcey , S. S. Dong , A. Dreuw , L. Freitag , L. M. Frutos , L. Gagliardi , F. Gendron , A. Giussani , L. González , G. Grell , M. Guo , C. E. Hoyer , M. Johansson , S. Keller , S. Knecht , G. Kovačević , E. Källman , G. L. Manni , M. Lundberg , Y. Ma , S. Mai , J. P. Malhado , P. Å. Malmqvist , P. Marquetand , S. A. Mewes , J. Norell , M. Olivucci , M. Oppel , Q. M. Phung , K. Pierloot , F. Plasser , M. Reiher , A. M. Sand , I. Schapiro , P. Sharma , C. J. Stein , L. K. Sørensen , D. G. Truhlar , M. Ugandi , L. Ungur , A. Valentini , S. Vancoillie , V. Veryazov , O. Weser , T. A. Wesołowski , P.-O. Widmark , S. Wouters , A. Zech , J. P. Zobel , and R. Lindh , “ Openmolcas: From source code to insight,” J. Chem. Theory Comput. 15, 5925–5964 (2019). 10.1021/acs.jctc.9b00532 31509407

[40] B. O. Roos , R. Lindh , P.-Å. Malmqvist , V. Veryazov , and P.-O. Widmark , “ Main group atoms and dimers studied with a new relativistic ANO basis set,” J. Phys. Chem. A 108, 2851–2858 (2004). 10.1021/jp031064+

[41] B. O. Roos , R. Lindh , P.-Å. Malmqvist , V. Veryazov , and P.-O. Widmark , “ New relativistic ANO basis sets for transition metal atoms,” J. Phys. Chem. A 109, 6575–6579 (2005). 10.1021/jp0581126 16834004

[42] F. Aquilante , L. Gagliardi , T. B. Pedersen , and R. Lindh , “ Atomic cholesky decompositions: A route to unbiased auxiliary basis sets for density fitting approximation with tunable accuracy and efficiency,” J. Chem. Phys. 130, 154107 (2009). 10.1063/1.3116784 19388736

[43] P.-Å. Malmqvist , K. Pierloot , A. R. M. Shahi , C. J. Cramer , and L. Gagliardi , “ The restricted active space followed by second-order perturbation theory method: Theory and application to the study of CuO_2_ and Cu_2_O_2_ systems,” J. Chem. Phys. 128, 204109 (2008). 10.1063/1.2920188 18513012

[44] K. Pierloot , “ The CASPT2 method in inorganic electronic spectroscopy: From ionic transition metal to covalent actinide complexes,” Mol. Phys. 101, 2083–2094 (2003). 10.1080/0026897031000109356

[45] M. Douglas and N. M. Kroll , “ Quantum electrodynamical corrections to the fine structure of helium,” Ann. Phys. 82, 89–155 (1974). 10.1016/0003-4916(74)90333-9

[46] B. A. Hess , “ Relativistic electronic-structure calculations employing a two-component no-pair formalism with external-field projection operators,” Phys. Rev. A 33, 3742 (1986). 10.1103/PhysRevA.33.3742 9897114

[47] P.-Å. Malmqvist and B. O. Roos , “ The CASSCF state interaction method,” Chem. Phys. Lett. 155, 189–194 (1989). 10.1016/0009-2614(89)85347-3

[48] P.-Å. Malmqvist , B. O. Roos , and B. Schimmelpfennig , “ The restricted active space (ras) state interaction approach with spin–orbit coupling,” Chem. Phys. Lett. 357, 230–240 (2002). 10.1016/S0009-2614(02)00498-0

[49] M. O. Krause and J. Oliver , J. Phys. Chem. Ref. Data. 8, 329–338 (1979). 10.1063/1.555595

[50] T. Lu and F. Chen , “ Multiwfn: A multifunctional wavefunction analyzer,” J. Comput. Chem. 33, 580–592 (2012). 10.1002/jcc.22885 22162017

[51] B. Thole , G. Van der Laan , J. Fuggle , G. Sawatzky , R. Karnatak , and J.-M. Esteva , “ 3d x-ray-absorption lines and the 3d^9^4f^n+ 1^ multiplets of the lanthanides,” Phys. Rev. B 32, 5107 (1985). 10.1103/PhysRevB.32.5107 9937719

[52] E. Stavitski and F. M. de Groot , “ The CTM4XAS program for EELS and XAS spectral shape analysis of transition metal L edges,” Micron 41, 687– 694 (2010). 10.1016/j.micron.2010.06.005 20637641

[53] R. V. Pinjari , M. G. Delcey , M. Guo , M. Odelius , and M. Lundberg , “ Cost and sensitivity of restricted active-space calculations of metal L-edge x-ray absorption spectra,” J. Comput. Chem. 37, 477–486 (2016). 10.1002/jcc.24237 26502979

[54] M. Kubin , M. Guo , T. Kroll , H. Löchel , E. Källman , M. L. Baker , R. Mitzner , S. Gul , J. Kern , A. Föhlisch , A. Erko , U. Bergmann , V. K. Yachandra , J. Yano , M. Lundberg , and P. Wernet , “ Probing the oxidation state of transition metal complexes: A case study on how charge and spin densities determine Mn L-edge x-ray absorption energies,” Chem. Sci. 9, 6813–6829 (2018). 10.1039/C8SC00550H 30310614 PMC6115617

[55] R. Manne , “ Chemical shifts in x-ray emission,” *Inner-Shell and X-Ray Physics of Atoms and Solids* ( Springer, 1981), pp. 699–706.

[56] R. M. Jay , J. Norell , S. Eckert , M. Hantschmann , M. Beye , B. Kennedy , W. Quevedo , W. F. Schlotter , G. L. Dakovski , M. P. Minitti , M. C. Hoffmann , A. Mitra , S. P. Moeller , D. Nordlund , W. Zhang , H. W. Liang , K. Kunnus , K. Kubiček , S. A. Techert , M. Lundberg , P. Wernet , K. Gaffney , M. Odelius , and A. Föhlisch , “ Disentangling transient charge density and metal-ligand covalency in photoexcited ferricyanide with femtosecond resonant inelastic soft x-ray scattering,” J. Phys. Chem. Lett. 9, 3538–3543 (2018). 10.1021/acs.jpclett.8b01429 29888918

[57] N. Dalleska , B. Tjelta , and P. Armentrout , “ Sequential bond energies of water to Na^+^ (3s0), Mg^+^ (3s1), and Al^+^ (3s2),” J. Phys. Chem. 98, 4191–4195 (1994). 10.1021/j100066a045

[58] S. Vancoillie , H. Zhao , V. T. Tran , M. F. Hendrickx , and K. Pierloot , “ Multiconfigurational second-order perturbation theory restricted active space (RASPT2) studies on mononuclear first-row transition-metal systems,” J. Chem. Theory. Comput. 7, 3961–3977 (2011). 10.1021/ct200597h 26598342

[59] I. Lindgren , “ Chemical shifts in x-ray and photo-electron spectroscopy: A historical review,” J. Electron Spectrosc. Relat. Phenom. 137–140, 59–71 (2004). 10.1016/j.elspec.2004.02.086

[60] L. Ramqvist , B. Ekstig , E. Källne , E. Noreland , and R. Manne , “ X-ray study of inner level shifts and band structure of TiC and related compounds,” J. Phys. Chem. Solids 30, 1849–1860 (1969). 10.1016/0022-3697(69)90253-4

[61] G. Leonhardt and A. Meisel , “ Determination of effective atomic charges from the chemical shifts of x-ray emission lines,” J. Chem. Phys. 52, 6189–6198 (1970). 10.1063/1.1672925

[62] J. Blomquist , B. Roos , and M. Sundbom , “ Use of the Mössbauer isomer shift and the x-ray emission Kα shift to estimate the effective electronic population of iron in complexes,” Chem. Phys. Lett. 9, 160–162 (1971). 10.1016/0009-2614(71)80213-0

